# Philippine Nurses Association of America’s Innovative Leadership Development Program: Community-Led Model for Nurse Leader Development and Chapter Governance

**DOI:** 10.1016/j.mnl.2025.102626

**Published:** 2026-03-23

**Authors:** Mary Dioise Ramos, Leli Pedro Drake, Ma Milani Zabala, Manny Ramos, Marlon Garzo Saria, Lorraine S. Evangelista

**Affiliations:** Associate Dean for Nursing Research, Scholarship and Science, Louisiana State University Health Sciences Center – New Orleans School of Nursing, New Orleans, Louisiana.; Professor Emerita, University of Colorado Anschutz Medical Campus, Aurora, Colorado.; Utilization Review Clinical Case Manager, University of California Davis Health Medical Center, Sacramento, California.; Professor of Nursing, Valencia College, Orlando, Florida.; President, Philippine Nurses Association of America, Inc, New Jersey.; Professor Emeritus, University of California Irvine Sue & Bill Gross School of Nursing, Irvine, California.

## Abstract

The Philippine Nurses Association of America developed the Innovative Leadership Development Program to strengthen chapter governance and build a culturally grounded pipeline of nurse leaders. A mixed-methods evaluation of the 2025 cohort (n=40) used standardized postcourse surveys and thematic analysis of open responses. Findings show high satisfaction and recommendation rates, self-reported gains in leadership, communication, project management, and financial stewardship, and strong value placed on SOAR analysis and the Project Management Plan; participants cited time constraints and requested more live interaction and coaching. Innovative Leadership Development Program is a replicable, community-led model for scalable leadership development.

Global health systems are becoming increasingly complex due to aging populations, the rise of chronic diseases, and technological advances, underscoring the need for strong nursing leadership to uphold quality, safety, and workforce resilience.^1,2^ Nurses trained abroad, including those from the Philippines, make up a large part of the global workforce but often encounter systemic barriers to advancing into leadership roles, such as credentialing challenges, limited access to culturally relevant development opportunities, and under-representation in executive positions.^3,4^ These issues highlight the importance of tailored leadership training that combines evidence-based skills with cultural awareness and organizational context.^1^

Professional organizations can bridge this gap by creating programs that integrate practical skills, mentorship, and community networks to enhance chapter governance and transform learning into tangible organizational results.^5^ These programs have been shown to improve nurse performance, strengthen organizational capacity, and promote clinical excellence across various settings when grounded in evidence-based leadership frameworks.^1^ The Philippine Nurses Association of America (PNAA) developed the Innovative Leadership Development Program (iLDP) to address documented leadership-preparation gaps among Filipino nurses in the United States and the diaspora, and to build a sustainable, culturally grounded pipeline of leaders. iLDP pairs a modular curriculum (communication, SOAR analysis, project management, fiscal stewardship), mentor-supported capstone project work, and cohort-based peer networks intended to produce timely, applied outcomes at the state/chapter level.

The purpose of this mixed-methods evaluation was to assess implementation fidelity, participant satisfaction, and perceived leadership outcomes of the 2025 iLDP cohort.

## Program Development and History

PNAA developed the iLDP to directly address recurring chapter challenges, such as variable governance capacity, inconsistent fiscal stewardship, and a lack of culturally congruent leadership training, aiming to translate individual growth into measurable chapter outcomes. Guided by a multistakeholder committee, the program emphasizes practical leadership and chapter influence over theory, focusing on domains such as strategic planning (using SOAR), communication, project management (via a Capstone Plan), and financial stewardship. Its multimodal, immersive instructional methods—combining asynchronous modules, live virtual “Café” chats, expert lectures, mentoring, and peer coaching—align with data suggesting project-based learning drives lasting behavioral change. Initiated as a pilot and expanded using a hybrid delivery model via chapter networks, iLDP implemented rapid feedback mechanisms to make continuous, incremental adjustments to the curriculum and delivery. For long-term success and replicability, the PNAA established an alumni-to-faculty pipeline and integrated chapter-embedded capstones to secure local ownership and sustain faculty capacity.

## METHODS

A convergent design was used to collect quantitative survey data and qualitative open-ended responses concurrently, then integrate findings to characterize program performance and identify actionable improvements.^6^ Participants were 40 PNAA members who completed the 2025 iLDP cohort. Eligibility required active PNAA membership and enrollment in the cohort; recruitment occurred via chapter announcements, national newsletters, and targeted outreach. The cohort included practicing nurses across multiple U.S. regions and roles (staff nurses, clinical educators, chapter officers).

A standardized postcourse survey administered via the program learning platform collected Likert-scale responses on program fidelity (consistency with promotional materials), content relevance, organization, effectiveness of virtual methods, attainment of specific learning outcomes (leadership perspective, communication, project management, financial stewardship), overall satisfaction, and likelihood to recommend. Items were adapted from established continuing professional development and leadership program evaluation tools to align with program objectives.^7^

Four open-ended prompts solicited participant reflections: Which iLDP content was most beneficial; factors affecting performance; how would you change if you knew then what you now know; and topics or structural changes recommended. Additional space allowed for Fellows comments and testimonials. Responses were exported verbatim for analysis. Curriculum maps, cohort timeline, capstone templates, faculty rosters, and iterative program modifications (post-module debrief notes) were reviewed to contextualize participant data and document program evolution.

Primary quantitative measures were item frequencies and proportions for each Likert item (agreement and satisfaction scales). A categorical summary identified the most frequently endorsed “most beneficial” content areas (e.g., project management, SOAR, fiscal responsibility). Enrollment and course platform records were employed to monitor completion and retention rates. Emerging themes regarding perceived impacts, barriers, and recommended enhancements were underscored by qualitative feedback.

### Data Analysis

Descriptive statistics, including frequencies and percentages, for each survey item were calculated using the exported dataset from the program (n = 40). No inferential testing was performed because the evaluation prioritized program description and improvement rather than hypothesis testing.

An inductive thematic analysis approach was used.^8^ Two analysts independently examined all responses to understand the data, created initial codes, and iteratively developed a thematic codebook. They examined their coding, addressed discrepancies to resolve issues, and synthesized overall concepts, choosing clear and representative quotations for illustration. Triangulation using program documents and quantitative trends helped develop the themes.

The evaluation amalgamated quantitative and qualitative findings to confirm convergence (e.g., survey support for Project Management aligning with the thematic focus in responses) and to uncover complementary insights (e.g., high satisfaction paired with requests for more live coaching). Credibility was bolstered through multiple analyst coding and the maintenance of a detailed audit trail. Quantitative data used all valid responses, excluding missing data on a case-by-case basis per item. Practical relevance and transferability were supported by including program artifacts during interpretation. As a program improvement activity under PNAA auspices, with voluntary, anonymous survey participation, the PNAA Committee on Research determined that Institutional Review Board review was not required.

## RESULTS

### Participant Characteristics and Engagement

Forty of 42 PNAA members completed the 2025 iLDP and the postcourse evaluation. Participants represented a range of chapter roles (staff nurses, clinical educators, chapter officers) and all 4 PNAA U.S. regions, and all 40 completed the required modules and the capstone Project Management Plan. Course platform logs confirmed full module completion and capstone submission for the cohort. These engagement data provide the foundation for interpreting both survey responses and qualitative reflections presented below.

### Program Fidelity, Satisfaction, and Learning Outcome Attainment

Table 1 summarizes participant ratings of program fidelity, satisfaction, and learning outcomes, with corresponding practice implications. Participants reported that the program matched its promotional materials and that disclosures (requirements and conflicts of interest) were clear at the outset, establishing strong program fidelity. Overall satisfaction was high: 75% reported being “very satisfied” and 85% were “extremely likely” to recommend the program. Faculty and mentors were rated as effective by most participants (72.5% strongly agreed), and nearly all respondents judged the content to be fair, balanced, and free of undisclosed bias or commercial influence. Although most participants affirmed the effectiveness of virtual delivery (67.5% strongly agree), qualitative responses flagged a desire for additional synchronous coaching and more live interaction to support capstone progress and application.

Self-reported attainment of the program’s stated learning outcomes was strong across domains: leadership perspective and communication outcomes were each strongly agreed by 90% of participants, project management by 85%, and financial stewardship by 87.5%. When asked which content area was most beneficial, participants most often selected “All / entire program” and “Project Management (capstone)” equally (each 27.5%), with leadership, fiscal responsibility, and organizational communication following (Table 2). These distributions indicate that core instructional investments, particularly the capstone Project Management Plan and SOAR-based strategic content, were perceived as both coherent with the program aims and immediately useful for chapter work. Table 3 summarizes the themes with vignettes and direct quotes from the participants.

### Qualitative Themes and Linkage to Quantitative Data

#### Strengthened Leadership Identity and Confidence

Participants observed a clear shift from volunteers focused solely on tasks to purposeful leaders who set priorities, gather resources, and assume official roles. They frequently mentioned reflective exercises and the leadership identity module as drivers of this transformation. Learners shared specific steps, such as drafting strategic priorities, joining governance committees, and viewing routine tasks as opportunities for leadership. These qualitative accounts align with high survey endorsement of leadership outcomes: 90.0% of participants “strongly agree” that the leadership learning outcome was met, and 80.0% “strongly agree” they met their personal objectives. The qualitative narrative explains how identity works, is translated into observable behavioral change, and provides a mechanism for the quantitative leadership outcome.

#### Rapid, Practical Application to Chapter Initiatives

Participants consistently highlighted the immediate usefulness of the SOAR framework and the capstone Project Management Plan in their current chapter work/leadership. Examples included converting outreach checklists into timelines with assigned owners, restructuring volunteer roles to improve turnout, and using the capstone template to launch a mentorship pilot with defined evaluation measures. This rapid application aligns with the quantitative prominence of project management: 27.5% named Project Management as the single most beneficial content, and 85.0% “strongly agree” that the project management outcome was met. The qualitative vignettes show the pathway from instruction to rapid operationalization, clarifying why the project management metrics are robust.

#### Enduring Mentorship and Peer Networks

Participants consistently described cohort bonds and mentor pairings as the primary relational mechanism sustaining program gains. Faculty coaches provided ongoing agenda reviews and practical feedback, while peer accountability groups continued meeting to share templates and troubleshoot implementation problems. These durable networks explain high coach-effectiveness ratings and program advocacy: 72.5% “strongly agree” faculty/coaches were effective, and 85.0% were “extremely likely” to recommend the program. The qualitative data illuminate how social scaffolding converts short-term learning into sustained chapter activity, leadership, and ongoing improvement.

#### Cultural Relevance and Resonance

Several participants noted that including culturally relevant examples, diaspora-specific scenarios, and chapter-integrated capstones made the curriculum feel genuine and better suited to local adaptation. When case studies covered family obligations, migration trends, and chapter politics, learners showed greater trust in the content and became more motivated to apply what they learned. Near-unanimous quantitative signals of trustworthiness reinforce this perceived fit: 100.0% confirmed clear disclosures and 97.5% rated the content as fair and balanced, with no undisclosed bias or commercial support. The qualitative descriptions illustrate how cultural resonance increased engagement and lowered translational friction.

#### Barriers and Improvement Opportunities

Despite overall positive ratings, a consistent subset of participants described time pressures, competing work and family responsibilities, and the need for more synchronous coaching as constraints on deeper engagement. The capstone, while valuable, was described as large and anxiety-provoking without staged feedback and targeted live support. These qualitative concerns map onto the survey pattern: most participants rated virtual teaching as effective (67.5% “strongly agree”), yet a minority gave neutral or lower ratings for organization and virtual effectiveness. Together, the data indicate that flexible asynchronous delivery succeeded for many learners but that deliberate, short-lived touchpoints and scaffolded capstone milestones would increase accessibility and completion confidence for those with heavier time constraints.

### Triangulated Interpretation Across Themes

**Figure 1.**
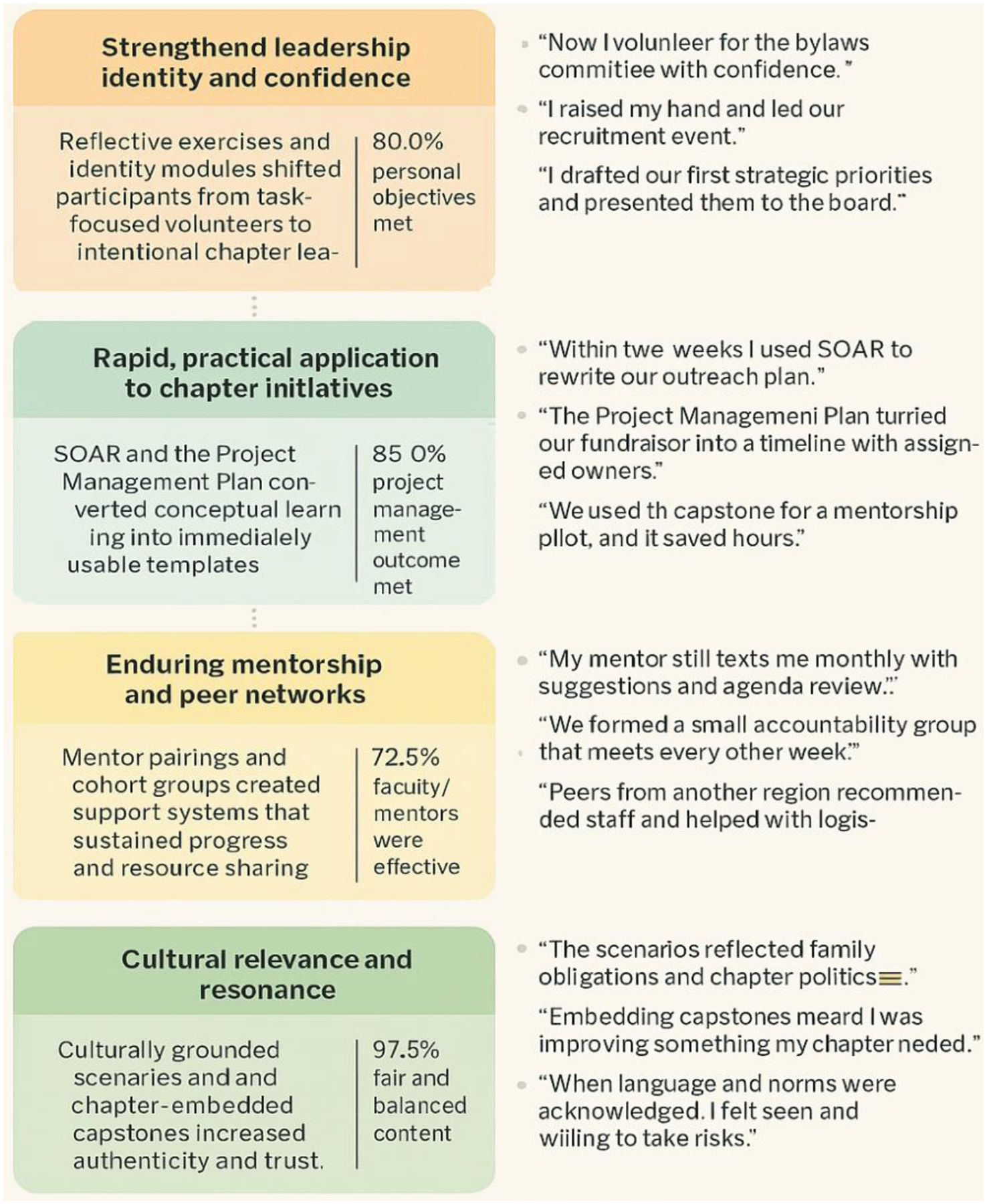
Thematic map showing 5 core themes from the iLDP evaluation with associated core ideas, key quantitative indicators, and representative participant reflection: themes are ordered to reflect both prevalence in qualitative data and supporting evidence. iLDP, Innovative Leadership Development Program.

Figure 1 illustrates how the qualitative themes and quantitative indicators form a coherent explanatory chain: identity work fostered confidence (leadership outcomes), practical tools enabled rapid application (project management metrics), and relational supports sustained implementation (mentor effectiveness and recommendation rates), all amplified by cultural fit (disclosure and balance metrics). The primary actionable gap—need for micro-coaching and phased capstone support—appears consistently across both data types, making it a high-priority refinement likely to increase equitable uptake and deepen program impact.

## DISCUSSION

The evaluation of the 2025 iLDP reveals that a culturally responsive, chapter-based leadership curriculum can lead to significant improvements in leadership identity, quick application of project management tools, sustainable mentorship networks, and greater learner satisfaction among Filipino American nurses. Participants’ stories of transitioning from task-oriented volunteers to purposeful leaders align with studies showing that organized reflection and identity development enhance leadership growth.^9,10^ In contrast to numerous hospital-centric leadership programs that associate identity work with unit metrics and managerial dashboards, the iLDP directly connected reflective exercises to chapter governance tasks and community engagement, thereby expediting the transition from insight to action; this contextual emphasis likely enhanced the robust endorsement of leadership outcomes noted in the evaluation.

The iLDP’s focus on practical, project-based learning quickly improved local chapter operations. Participants reported turning SOAR analyses and capstone Project Management Plans into timelines, role matrices, and pilot evaluations within weeks—a pattern supported by systematic reviews showing that ongoing professional development with tangible deliverables improves practical application.^7^ Training science corroborates this methodology: programs that integrate explicit objectives, practice opportunities, and feedback yield more substantial and enduring outcomes than passive instruction.^11^ iLDP stands out from several internal organizational pipelines by localizing initiatives within chapter contexts, which typically face fewer institutional clearances and can iterate more quickly than hospital programs constrained by formal governance and regulatory frameworks. The variation in where execution occurs can be particularly important when the goal is a clear, short-term impact for volunteer-driven initiatives.

Deliberate paired coaching and peer networks evolved into important mechanisms that supported program advancement. Participants reported continuous coach check-ins and interchapter accountability groups that persisted in sharing templates, resolving logistical issues, and offering social support postcourse completion. Relational scaffolds are well documented in the literature as catalysts for career progression, employee retention, and lasting practice transformation.^12^ The horizontal, cross-regional coaching structure of iLDP contrasts with the employer-embedded approaches common in Magnet-aligned pipelines, where mentorship is usually integrated into managerial hierarchies. The horizontal setup may enhance portability and continuity when participants move between jobs or locations, supporting sustained leadership development outside any specific workplace environment.^1^

Cultural relevance functioned as an important amplifier across outcomes. Participants reported that diaspora-specific case scenarios and chapter-embedded capstones made the content feel authentic, increased trust (personal and professional), and reduced translational friction. The benefits of culturally tailored curricula are well supported by peer-reviewed work showing that culturally congruent interventions increase participant engagement and implementation success.^13,14^ Many mainstream leadership programs treat culture as an optional module; iLDP’s approach of making cultural context a core design element appears to have strengthened motivation and relevance for this cohort and may be an especially potent strategy for programs serving diaspora or other identity-defined professional communities.

Although the overall results were positive, participants highlighted challenges, indicating that practical adjustments are needed. Time constraints, conflicting clinical and familial obligations, and demands for increased synchronous coaching persisted. This pattern aligns with blended learning research demonstrating that asynchronous content enhances accessibility, while concise, focused synchronous interactions—such as microcoaching, live clinics, and staged feedback—-improve completion rates, confidence, and the application of complex tasks.^15,16^ Because PNAA members typically engage in chapter work alongside full-time clinical roles without protected time, redistributing a small portion of asynchronous hours to short live touchpoints is a practical design adaptation likely to enhance equitable participation.

Comparing iLDP to Magnet-aligned internal leadership pipelines clarifies both shared mechanisms and meaningful differences. Both program types employ identity work, experiential assignments, mentorship, and multimodal delivery, and both aim to develop leaders who can change practice. Magnet-aligned pipelines, however, usually embed projects in unit-level clinical performance metrics and place mentorship within employer structures, which can facilitate institutional support for implementation but may also slow iteration due to organizational approvals and competing priorities.^17,18^ iLDP’s chapter embedded focus and cultural centrality appear to enable faster, more visible application in community contexts and to create cross-chapter networks that persist independently of employers. These contrasts suggest a complementary relationship rather than a hierarchical one: iLDP-style programs may accelerate early adoption and leadership identity formation in community or association settings, whereas Magnet-aligned pipelines may be better suited for embedding long-term clinical performance metrics within health system quality frameworks. Lessons learned from this evaluation emphasize 4 design principles that appear to have driven impact: anchor identity work to locally meaningful tasks; require applied, chapter-relevant deliverables; formalize horizontal mentorship and peer networks for postcourse sustainment; and embed cultural context centrally rather than treat it as an add-on. Implementing concise microcoaching clinics and structured capstone checkpoints within a blended framework mitigates a persistent obstacle without significantly increasing participants’ total time commitment.

### Implication for Nurse Leaders and Professional Organizations

Linking leadership identity development to relevant chapter tasks enhanced participants’ preparedness to undertake formal positions and convert personal insights into concrete organizational actions. This connection seemed more direct and effective than in several employer-sponsored leadership programs.^9,10^ The execution of a practical, chapter-based capstone aligned with the Project Management Plan and SOAR framework allowed for quick skill application and immediate successes that enhanced learning and motivation, supporting research that shows project-based professional development improves practical knowledge.^7,11^

The establishment of horizontal mentorship and peer accountability networks fostered enduring, transferable social frameworks that facilitated sustained implementation and diminished reliance on employer infrastructure, consistent with literature on communities of practice and the advantages of structured mentoring.^12^ Incorporating cultural relevance as a fundamental design principle—rather than a supplementary feature—enhanced authenticity, trust, and acceptance among diaspora and identity-defined professional groups.^13,14^ Ultimately, incorporating concise synchronous microcoaching and incremental capstone milestones into primarily asynchronous curriculum can alleviate time constraints for practicing clinicians while improving feedback, confidence, and engagement.^16^ Healthcare organizations can strengthen leadership by using iLDP’s project-based capstones, micro-coaching clinics, and mentorship networks. Incorporating reflective identity work and culturally relevant case studies into hospital or academic programs may accelerate progress for minority and internationally educated nurses. Incorporating these tactics into Magnet-aligned or shared-governance frameworks can create a dual path—one that promotes community-based leadership through groups like PNAA and the other that strengthens institutional leadership within clinical entities.

## CONCLUSION

The 2025 iLDP evaluation shows that a culturally centered, chapter-embedded leadership program yields immediate, measurable gains in leadership identity and project implementation, all while achieving high participant satisfaction. PNAA succeeded by linking reflective identity work to local tasks, mandating a project-based capstone, formalizing cross-chapter mentorship, and embedding cultural relevance throughout. These design features—localized capstones, simplified tools, micro-coaching, and formalized mentorship—are practical, scalable, and transferable. Other diaspora- or identity-based organizations can replicate these core elements, with minor adaptations to accommodate synchronous time constraints, to rapidly enhance leader development and organizational capacity.

## Figures and Tables

**Table 1.** Program Evaluation Summary: Fidelity, Satisfaction, and Learning Outcomes (n = 40)

Domain / Item	% Strongly Agree	Key Finding or Description	Implications for Leadership Practice
*Program fidelity*			
*Program consistent with advertised materials*	82.5%	Content matched promotional objectives and met learning outcomes.	Demonstrates strong alignment between program design and participant expectations.
*Requirements and conflicts of interest disclosed at the start*	100.0%	Transparency was maintained throughout the program.	Builds trust and credibility with learners.
*Content is fair, balanced, and free from bias or commercial influence*.	97.5%	No reports of undisclosed bias or commercial support.	Reinforces an ethical and culturally safe learning environment.
*Teaching effectiveness and satisfaction*			
*Faculty and mentors are effective*.	72.5%	Faculty and mentors provided high-quality guidance and feedback.	Highlights the value of mentorship and peer accountability in sustaining learning.
*Virtual teaching effective*	67.5%	The majority were satisfied with hybrid virtual delivery; requests for more live coaching were noted.	Support adding micro-coaching sessions to enhance engagement.
*Overall satisfaction*	75.0%	Three-quarters were “very satisfied.”	Indicates high overall program approval.
*Likelihood to recommend the program*	85.0%	Participants would recommend iLDP to peers.	Suggests strong advocacy and program reputation among nurse leaders.
*Learning outcome attainment*			
*Appraise leadership at individual, organizational, and policy levels*.	90.0%	Participants gained insight into multi-level leadership functions.	Reinforces the effectiveness of reflective leadership identity work.
*Analyse a systematic approach to communication*.	90.0%	Improved understanding of communication strategies for chapter governance.	Underscores the need to continue prioritizing communication in leadership training.
*Create a project management plan using PM principles*.	85.0%	The Capstone Project Management Plan is highly valued.	Confirms the practical impact of project-based learning.
*Evaluate financial management principles*.	87.5%	Increased confidence in fiscal stewardship and budgeting.	Supports integration of fiscal modules into ongoing leadership curricula.

**Table 2.** Most Beneficial Content (Single Choice) (n = 40)

Content Area	n	%
*All/entire program*	11	27.5%
*Project management (capstone)*	11	27.5%
*Leadership in complex organizations*	8	20.0%
*Fiscal responsibility*	6	15.0%
*Organizational communication*	4	10.0%

**Table 3.** Themes With Vignettes and Participant Quotes

Theme	Vignette	Direct Quotes from Participants.
*Strengthened leadership identity and confidence*	Reflective exercises and the leadership identity module shifted participants from task executors to intentional chapter leaders who initiate strategy and accept formal roles.	“I always thought chapter work was ‘busy work’ until the identity module helped me name my leadership strengths.” “After iLDP, I raised my hand and led our recruitment event; I finally felt ready.” “Before the program, I deferred to others; now I drafted our first strategic priorities and presented them to the board.”
*Rapid, practical application to chapter initiatives*	SOAR and the Project Management Plan converted conceptual learning into immediately usable templates for events, volunteer coordination, and pilot projects.	“Within 2 weeks, I used the SOAR framework to rewrite our outreach plan, and volunteers showed up.” “The Project Management Plan turned our fundraiser from a checklist into a timeline with assigned owners; we hit our targets.” “We used the capstone template for a mentorship pilot; it saved hours of planning and clarified evaluation measures.”
*Enduring mentorship and peer networks*	Mentor pairings and cohort accountability groups created ongoing support systems that sustained project work, troubleshooting, and resource sharing beyond the course end.	“My mentor still texts me monthly with suggestions and a quick review of my meeting agenda.” “We formed a small accountability group that meets every other week to share templates and lessons learned.” “When I needed volunteers for a screening, peers from another region recommended staff and helped with logistics.”
*Cultural relevance and resonance*	Culturally grounded scenarios and chapter-embedded capstones increased authenticity, trust, and willingness to adapt tools to local chapter realities.	“The case scenarios reflected our realities—family obligations and chapter politics—so solutions felt realistic.” “Embedding capstones in chapter work meant I wasn’t doing ‘homework’ in isolation; I was improving something my chapter really needed.” “When language and cultural norms were acknowledged in examples, I felt seen and more willing to take risks with leadership behaviors.”
*Barriers and improvement opportunities*	Time constraints, competing responsibilities, and the need for staged capstone checkpoints and brief live coaching were the primary barriers to deeper engagement for a subset of participants.	“I loved the content, but juggling 12-hour shifts and family made deadlines tight; a few live check-ins would have helped.” “The capstone felt big—if there had been staged feedback points, I would have iterated faster and with less stress.” “Virtual modules were efficient, but a brief mid-program coaching clinic would have helped translate concepts into our chapter realities.”

## KEY POINTS

- Leaders must invest in culturally tailored training to drive engagement and trust, mirroring the high satisfaction achieved when content resonates with diaspora experiences.
- Leaders should prioritize skill transfer by making high-value tools, like SOAR analysis and the capstone plan to rapidly forge effective project managers and leaders.
- Leaders must strategically embed synchronous coaching and phased milestones to support complex deliverables and ensure equitable completion
